# Epidemiological and phylogenetic analysis for non-B subtypes of human immunodeficiency virus type 1 in Busan, Korea

**DOI:** 10.1038/s41598-021-94794-1

**Published:** 2021-08-06

**Authors:** Jeong Eun Lee, Soon Ok Lee, Shinwon Lee, Sohee Park, Hyung-Hoi Kim, Kyung-Hwa Shin, Jin Suk Kang, Sun Hee Lee

**Affiliations:** 1 Division of Infectious Disease, Department of Internal Medicine, Pusan National University School of Medicine and Medical Research Institute, Pusan National University Hospital, 179 Gudeok-ro, Seo-gu, Busan, 49241 Republic of Korea; 2 Deparment of Laboratory Medicine, Pusan National University School of Medicine, Busan, Republic of Korea; 3 Biomedical Informatics Unit, Pusan National University School of Medicine, Busan, Republic of Korea; 4 Division of Infectious Disease, Department of Internal Medicine, Inje University College of Medicine, Busan, Republic of Korea

**Keywords:** HIV infections, Viral epidemiology

## Abstract

Recent data on non-B subtypes’ epidemiology among patients infected with human immunodeficiency virus-1 (HIV-1) in Korea are lacking. We aimed to assess the changing trends in the epidemiology of non-B subtypes of HIV-1 in Korea using phyloepidemiological analyses. We analyzed the demographic records and sequencing data obtained from genotypic drug resistance tests between 2005 and 2019 from 517 patients infected with HIV attending a tertiary care hospital in Busan, Korea. Subtyping and phylogenetic analyses with reference sequences were performed. Additionally, transmission clusters were identified via maximum-likelihood trees. Non-B subtypes accounted for 21.3% of the 517 sequences. CRF01_AE (52.7%) was the most common non-B subtype, followed by CRF02_AG (16.4%), A1 (11.8%), and C (5.5%). The prevalence of non-B subtypes decreased from 36.4 to 13.4% by 2009, while it increased to 27.4% between 2015 and 2019. Among patients with non-B subtypes, the proportion of overseas sailors decreased from 66.7 to 7.5%; contrarily, the proportion of men-who-have-sex-with-men (MSM) increased from 0 to 46.9% over the study period. We identified 8 transmission clusters involving non-B subtypes, with sizes ranging from 2 to 4 patients, including 3 clusters containing MSM. Our results highlight the changes in the epidemiological trends of non-B subtypes of HIV-1 in Korea.

## Introduction

Since the first case of the human immunodeficiency virus (HIV) infection was reported in Korea in 1984, the number of patients with HIV has steadily increased^1^. As of December 2019, the cumulative number of patients with HIV infection in Korea, including non-ethnic Koreans, was 18,724, and more than 1000 new HIV cases have been reported annually since 2013^2^. With the increase in the number of cases, the epidemiological characteristics of HIV have also changed in Korea^3^. After the acquired immunodeficiency syndrome (AIDS) Prevention Law was enacted in December 1987, active mass mandatory screening tests for HIV were conducted for certain groups, including overseas sailors (OSs), sex workers and workers in “hygiene-related jobs” such as food factories, hotels, and inns^1,3,4^. In the early period of the HIV epidemic in Korea, HIV infections were detected in these groups until the end of this screening in 1993^1,3^. OSs were screened for HIV infection at quarantine laboratories on returning home^1^. Among the 876 infected individuals reported from 1985 to 1998, OSs accounted for 18.4%^1^. Previous studies have shown that 80% of patients in these groups and their partners were infected with non-B subtype viruses^5,6^.

Since then, the target population for compulsory HIV testing has been gradually minimized to sex workers, blood donors, military recruits and prisoners, and voluntary testing at hospitals and anonymous testing at public health centers were encouraged^3,4^. OSs are still required to undergo a general health check-up including HIV test every 2 years under the Seafarer’s Act in Korea^7^.

The number of cases infected domestically began to increase in the 1990s^1,3^. The annual number of patients newly diagnosed with HIV has been steadily increasing and exceeded 500 in 2003, and 1000 in 2013^2^. The proportion of Men-who-have-sex-with-men (MSM) has been increasing and the age at diagnosis has been decreasing^3^. According to the HIV surveillance data from the Korean Centers for Disease Control and Prevention (KCDC) in 2000, heterosexual contact was a major mode of HIV transmission and MSM only accounted for 28%^8^. In a recent nationwide HIV cohort study of Korea that included 1442 patients between 2006 and 2016, MSM accounted for 60.4%^9^. The proportion of MSM was higher in younger age groups, 71.5% in 18–29 years and 92.9% in 18–19 years, respectively^3^. Currently, more than 80% of the HIV epidemic in Korea is attributed to subtype B^6,10^. A recent nationwide study demonstrated that subtype B was identified in 93.1% of 927 samples collected between 1999 and 2012^6^. Furthermore, several previous studies reported that the Korean HIV epidemic was composed of a distinct monophyletic clade, Korean clade B (B^k^), rather than the pandemic form of subtype B^6,10–12^. B^k^ was estimated to have emerged less than 10 years after the introduction of subtype B in Korea. The B^k^ epidemic rapidly increased until the early 1980s, followed by modest growth until the 1990s, after which there was a plateau^10,13^. MSM are increasingly identified as the major high-risk groups in the B^k^ epidemic in Korea^10^. These findings showed that the HIV-1 subtype distribution in association with changes in the socio-demographic determinants of the HIV infection has dynamically changed over time in Korea. While the epidemiology of HIV-1 subtype B in Korea is well established, little is known about HIV-1 non-B subtypes.

Therefore, to gain a deeper understanding of HIV-1 non-B subtype epidemics in Korea, we conducted a molecular epidemiological study using available sequence data collected from genotypic drug resistance tests between 2005 and 2019. We aimed to evaluate the changing trends in the epidemiology of HIV-1 non-B subtypes, identify transmission clusters, and characterize the people in those clusters in Korea^14^.

## Results

### Study population

The demographic and clinical characteristics of the 517 patients are summarized in Table 1. Most patients included in the study were ethnic Koreans (96.5%) and male (90.1%), with a median age of 42.0 (31.0–52.0) years. MSM transmission route was most common (56.5%), and median CD4 cell counts at the sampling time were 234.5 (interquartile range [IQR], 77.3–399.3) cells/µL. Overall, 78.9% of subjects (n = 408) were antiretroviral therapy (ART)-naive; the remaining 21.1% (n = 109) were ART-experienced. Subtype B infections were predominant in the total study population at 78.7%. Non-B subtype strains were identified in 110 patients (21.3%). Several demographic features differed between patients with HIV-1 non-B subtypes and those with subtype B. Women were more likely to harbor non-B infections (31.8% of all non-B) than B infections (3.9% of all B). Only 1% of B but 12.7% of non-B infections were found among patients of non-Korean ethnicity. The proportion of patients categorized as former OSs or overseas workers (OWs) was significantly higher for non-B subtype infection than for subtype B infection (20.0% vs. 3.9%, *P* ≤ 0.001, 18.2% vs. 2.7%, *P* ≤ 0.001, respectively). On the contrary, MSM were more frequently infected with subtype B than the non-B subtype (67.1% vs. 17.3%).

**Table 1:** Demographic and clinical characteristics of the study population according to the B/Non-B subtype.

Variables	Total N = 517	Subtype B (n = 407)	Subtype non-B (n = 110)	*P*-value
Median age, years	42.0 (31.0–52.0)	42.0 (31.0–51.0)	44.0 (30.0–55.0)	0.471
**Sex**				< 0.001
Male	466 (90.1)	391 (96.1)	75 (68.2)	
Female	51 (9.9)	16 (3.9)	35 (31.8)	
**Ethnicity**				< 0.001
Koreans	499 (96.5)	403 (99.0)	96 (87.3)	
Non-Koreans	18 (3.5)	4 (1.0)	14 (12.7)	
**Marriage**				0.001
Unmarried	301 (58.2)	254 (62.4)	47 (42.7)	
Ever-married	208 (40.2)	147 (36.1)	61 (55.5)	
Unknown	8 (1.5)	6 (1.5)	2 (1.8)	
**Route of transmission**				< 0.001
Heterosexual	207 (40.0)	122 (30.0)	85 (77.3)	
MSM	292 (56.5)	273 (67.1)	19 (17.3)	
Unknown	18 (3.5)	12 (2.9)	6 (5.4)	
**Occupation**				
Ever OSs	38 (7.4)	16 (3.9)	22 (20.0)	< 0.001
Ever OWs	31 (6.0)	11 (2.7)	20 (18.2)	< 0.001
Former FSWs	6/51 (11.8)	2/16 (12.5)	4/35 (11.4)	1.000
Median CD4 cell counts (/µL)	234.5 (77.3–399.3)	219.5 (80.3–388.0)	261.0 (63.8–470.8)	0.261
**ART**				0.634
ART-naïve	408 (78.9)	323 (79.4)	85 (77.3)	
ART-experienced	109 (21.1)	84 (20.6)	25 (22.7)	
**Year of HIV diagnosis**				0.006
1988–1999	33 (6.4)	21 (5.2)	12 (10.9)	
2000–2004	46 (8.9)	34 (8.4)	12 (10.9)	
2005–2009	112 (21.7)	97 (23.8)	15 (13.6)	
2010–2014	180 (34.8)	149 (36.6)	31 (28.2)	
2015–2019	146 (28.2)	105 (26.0)	40 (36.4)	

Values are expressed as number (%) or number (IQR).

*HIV* human immunodeficiency virus, *MSM* men-who-have-sex-with-men, *OS* overseas sailor, *OW* overseas worker, *FSW* female sex worker, *CD4* cluster of differentiation 4, *ART* antiretroviral therapy, *IQR* interquartile range.

### Epidemiological trends of non-B subtypes of HIV-1

**Figure 1:**
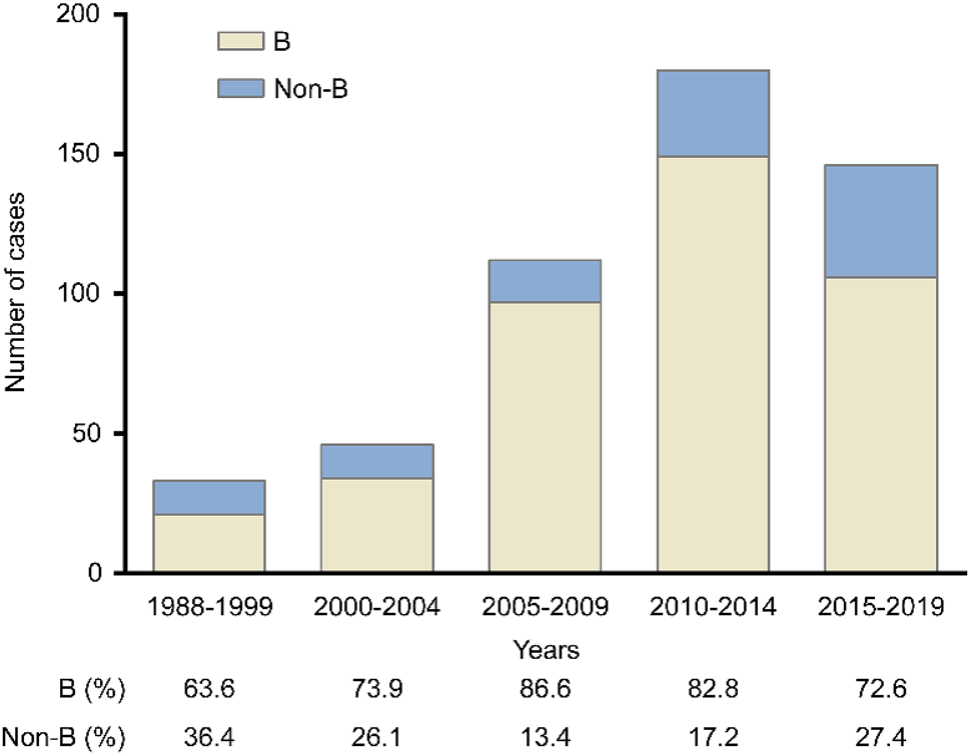
Changes in the proportion of HIV subtypes B and non-B subtypes among 517 patients with HIV infection over time, stratified by the year of HIV diagnosis.

**Figure 2:**
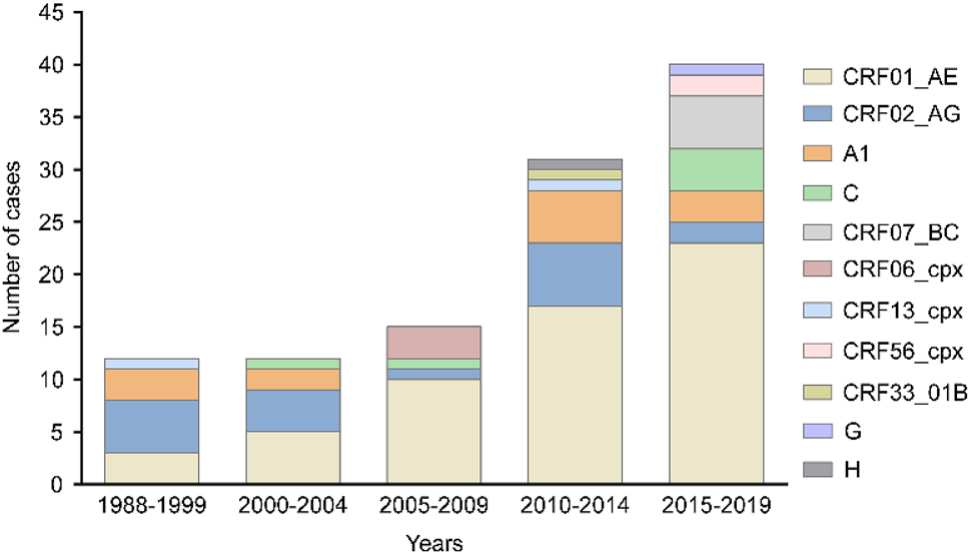
Changes in the distribution of non-B HIV-1 subtypes among 110 patients with HIV infection over time, stratified by the year of HIV diagnosis.

**Figure 3:**
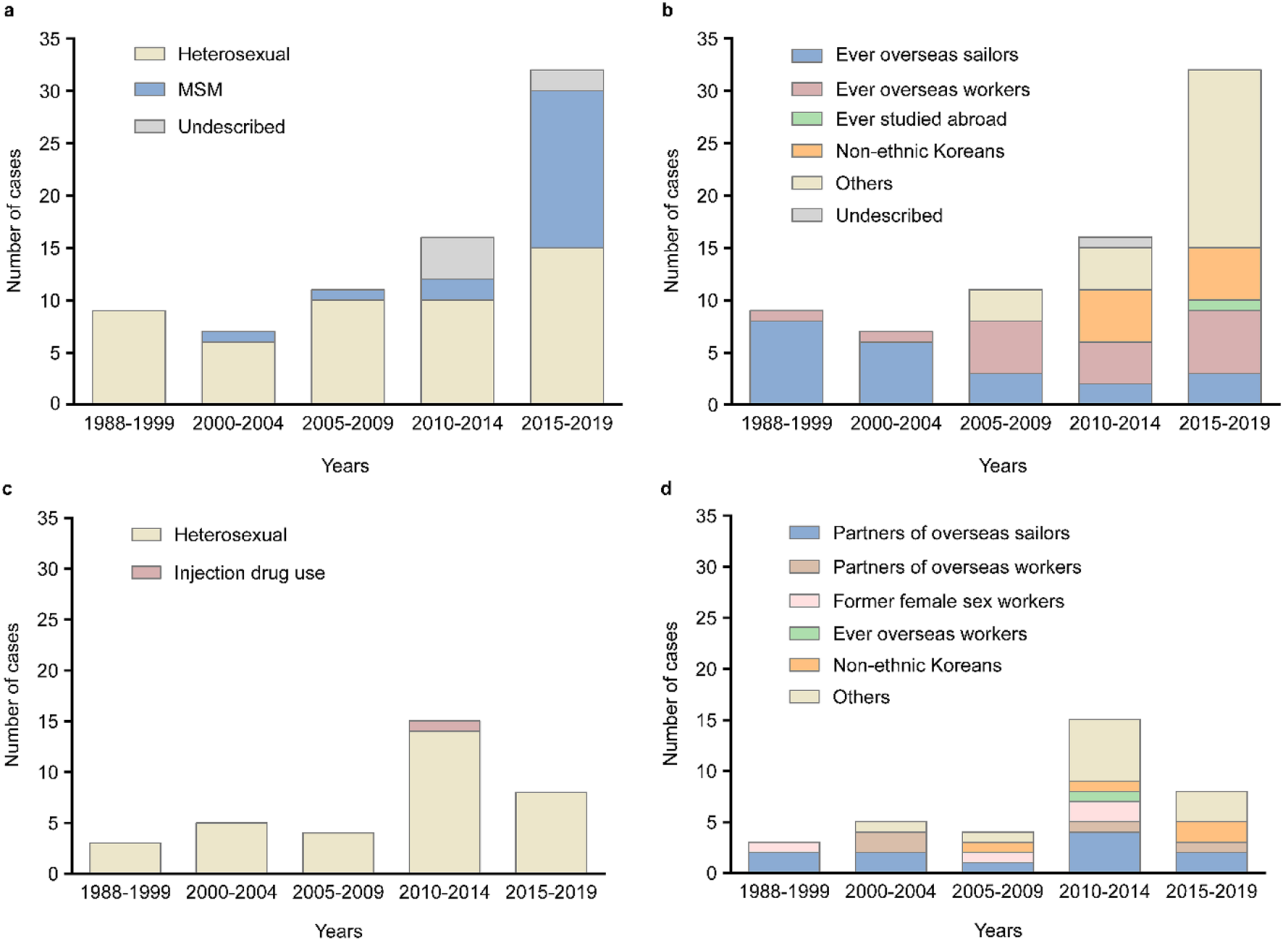
Changes in demographic characteristics of 110 patients with HIV infection harboring non-B subtypes, stratified according to the year of HIV diagnosis. (**a**) Men, route of infection (**b**) men, risk groups for the acquisition of non-B HIV-1 subtypes (**c**) women, route of infection (**d**) women, risk groups for acquisition of non-B HIV-1 subtypes.

Trends over time showed that the proportion of non-B subtype infection decreased from 36.4% in 1988–1999 to 13.4% in 2005–2009, and then increased to 17.2% and 27.4% in 2010–2014 and 2015–2019, respectively (Fig. 1). Among the 110 non-B subtype infections, CRF01_AE (52.7%) was the most prevalent, followed by CRF02_AG (16.4%), A1 (11.8%), C (5.5%), CRF07_BC (4.5%), CRF06_cpx (2.7%), CRF13_cpx (1.8%), CRF56_cpx (1.8%), CRF33_01B (0.9%), G (0.9%), and H (0.9%) (Fig. 2). The subtype distribution within non-B subtypes had also changed over time. Before 2000, CRF02_AG was the most commonly identified subtype, followed by A1 and CRF01_AE. Since then, the CRF01_AE diagnoses continuously increased from 25.0% in 1985–1999 to over 57.5% in 2015–2019 (Fig. 2). Demographic changes were also observed over time (Fig. 3). Among the 75 men infected with non-B subtypes, the proportion of MSM significantly increased in recent years, from 12.5% in 2010–2014 to 46.9% in 2015–2019 (*P* = 0.007) (Fig. 3a). The proportion of OWs (*P* = 0.504) and non-ethnic Koreans (*P* = 0.259) also tended to increase, but there was no statistical significance (Fig. 3b). In contrast, the proportion of OSs decreased from 66.7% in 1985–1999 to 7.5% in 2015–2019 (*P* ≤ 0.001) (Fig. 3b). Approximately 40% of women were partners of OSs (31.4%) or OWs (11.4%) (Fig. 3d).

**Figure 4:**
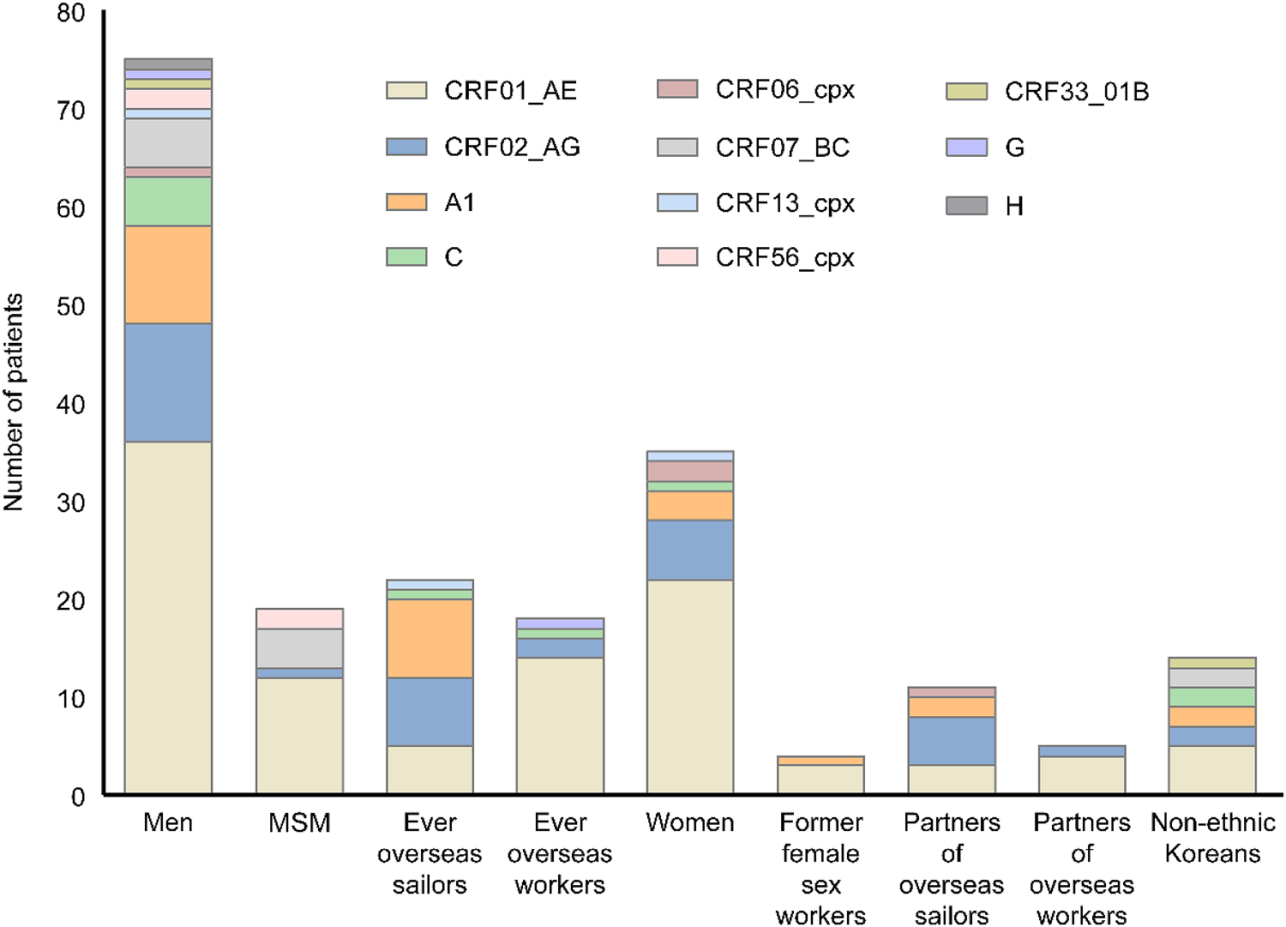
Changes in the distribution of non-B HIV-1 subtypes among 110 patients with HIV infection, stratified by the risk groups for HIV infection.

There was a difference in the subtype distribution according to the demographic subgroup (Fig. 4). In both men and women, the most frequent non-B subtype was CRF01_AE (52.7%), followed by CRF02_AG (16.4%) and A1 (11.8%). Among MSM, CRF01_AE (63.2%), CRF07_BC (21.1%), and CRF56_cpx (10.5%) were the predominant non-B subtypes, accounting for 94.7% of 19 non-B strains found in MSM. Among OSs, A1 was found in 36.4% of patients, followed by CRF02_AG (31.8%) and CRF01_AE (22.7%), while CRF01_AE accounted for 77.8% in OWs.

### Phylogenetic clusters

**Figure 5:**
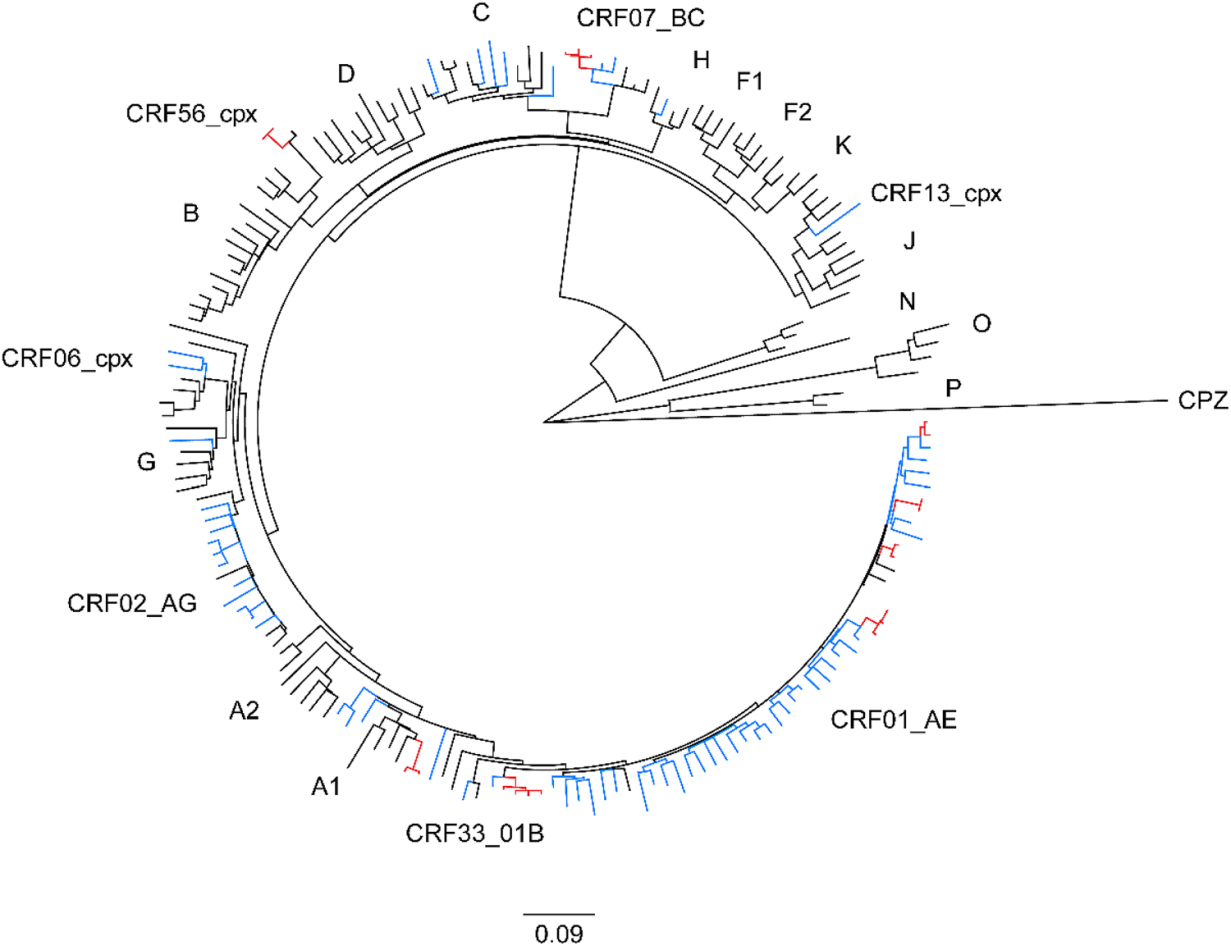
Maximum-likelihood phylogenetic tree of HIV-1 non-B subtype *pol* sequences from 83 antiretroviral therapy-naïve HIV-infected patients in Busan, Korea and 104 references from the Los Alamos National Laboratory (LANL) HIV database (http://www.hiv.lanl.gov). The maximum likelihood tree was constructed using MEGA version 10.1.8 (https://www.megasoftware.net) under the GTR + G + I model with 1000 bootstrap replicates. The black branch color represents the reference sequences, and the blue branch color indicates the study sequences, with the 8 transmission clusters highlighted in red.

**Figure 6:**
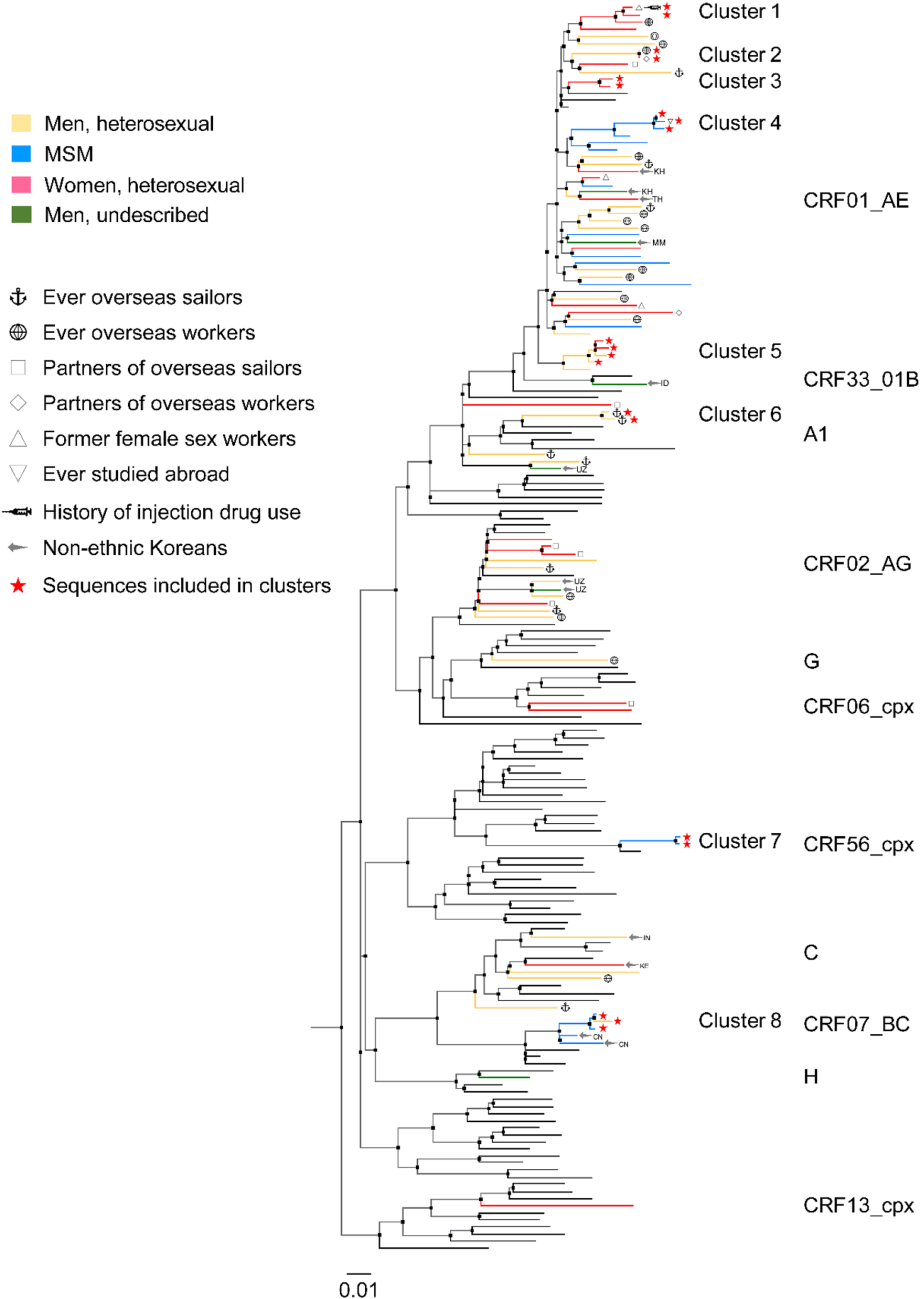
Transmission clusters in the maximum likelihood phylogenetic tree. The maximum likelihood tree was constructed using MEGA version 10.1.8 (https://www.megasoftware.net) under the GTR + G + I model with 1000 bootstrap replicates. The black branch color represents the reference sequences, and the colored branches represent transmission risk groups. Diameters of internal node circles reflect bootstrap values. Anchor emojis depict overseas sailors, and the globes with meridians represent overseas workers. Squares indicate the partners of overseas sailors, and diamonds indicate the partners of overseas workers. Triangles indicate female sex worker, and reverse triangles indicate subjects who had ever studied abroad. Arrows and two-letter country codes indicate foreigners. The syringe emoji indicates an injection drug user. Sequences included in clusters are indicated by red stars and their cluster names. *CRF* circulating recombinant form, *CN* China, *ID* Indonesia, *IN* India, *KE* Kenya, *KH* Cambodia, *MM* Myanmar, *TH* Thailand, *UZ* Uzbekistan.

Phylogenetic analysis was performed in 83 ART-naive patients with non-B subtypes, and 8 small clusters were identified with sizes ranging from 2 to 4 patients (Figs. 5 and 6). In total, 24.1% (20/83) of the patients were included in clusters. Among them, 7 (35%, mean age 26 years) were MSM, 7 (35%, mean age 35 years) women, and 6 (30%, mean age 38 years) heterosexual men. MSM (7/18, 38.9%) were more commonly included in clusters than women (7/25, 28%) and heterosexual men (6/34, 17.6%). Of the 8 clusters, 5 (62.5%) belonged to CRF01_AE and the remaining 3 clusters were distributed among A1, CRF56_cpx, and CRF07_BC. Five (62.5%) clusters included pairs, and 3 (37.5%) clusters included 3–4 patients. Four (50%) clusters included only men, 2 (25%) included only women, and 2 (25%) contained sequences from both men and women. Of the 2 male/female clusters, one cluster (cluster 2) included a married couple and the other (cluster 5) included 2 heterosexual men and 2 women whose epidemiological connections were unidentified. In some clusters, the composition was dominated by a single risk factor. Two (25%) clusters, cluster 4 (CRF01_AE) and cluster 7 (CRF56_cpx), comprised of sequences only from MSM. Cluster 6 (A1) comprised of sequences from OSs. In contrast, some clusters had multiple risk factors. Cluster 1 (CRF01_AE) included 2 women and one of them had a history of being and female sex worker (FSW) and an injection drug user (IDU), but the other did not. A mixed cluster containing sequences from individuals with different sexual behaviors, including 2 MSM and a heterosexual man, was also found for CRF07_BC (cluster 8). A detailed description is provided in Fig. 6.

## Discussion

The southeastern region of Korea, including Busan, the largest international port city in Korea, was one of the most affected areas of the HIV-1-related early epidemic in Korea. Many HIV infections have been identified in OSs and their spouses^3^. Of the 103 HIV infections newly diagnosed in this region between 1988 and 1997, 81% of cases were transmitted through heterosexual contacts, and 80% of them were OSs or their spouses^15^. In a previous phylogenetic analysis of *nef* gene from 102 OSs and 14 of their spouses enrolled between 1985 and 1998, non-B subtypes accounted for 80% of the cases, and among them, CRF02_AG was the most common, followed by CRF01_AE and A1^5,6^. With the change in the epidemic pattern in Korea, the proportion of MSM in this region also increased over time, from 12.6% in 1990–1997 to 41.2% in 2002–2011^15^. In this study, approximately 80% of HIV-1 sequences were of subtype B, and MSM accounted for 67% of them, which is broadly in line with previous epidemiological studies in Korea^3,9,15^.

However, we found that the epidemiological profile of HIV-1 non-B subtype infection in this region has been slowly changing over the decades. Trends showed that the proportion of non-B subtypes among the newly diagnosed HIV-1 patients initially decreased from 36.4% in 1988–1999 to 13.4% in 2005–2009 as the number of OSs and their partners gradually decreased, and the number of MSM relatively increased over time. The recent upward trend in the proportion of non-B subtypes may be partially explained due to the increasing numbers of OWs and immigrant workers from endemic countries with non-B subtypes. However, we also found that the proportion of MSM significantly increased in recent years, from 12.5% in 2011–2014 to 46.9% in 2014–2019.

In the phylogenetic analysis, MSM were more commonly included in clusters than heterosexual men and women. MSM in clusters are more likely to be younger and recently diagnosed with HIV than heterosexual men and women. Of the 18 MSM with non-B subtypes, 7 (38.9%) were included in 3 small clusters, with sizes ranging from 2 to 3 patients. Cluster 4 (CRF01_AE) included 3 young MSM and 2 of them reported homosexual contacts in Korea. Overall, 75% (9/12) of MSM with CRF01_AE were recently diagnosed with HIV between 2015 and 2019 and the majority of them reported no history of sexual activity overseas. These findings suggest that CRF01_AE is recently increasing in the MSM population in this region. Recent studies in Asian countries have shown that CRF01_AE strains have increased in their MSM population^16^. Recent studies in China have shown a dramatic shift in genotype distribution from subtype B to CRF01_AE among MSM^17,18^. Therefore, our findings underscore the importance of strengthening epidemiological surveillance in this region.

We also identified a small transmission pair harboring CRF56_cpx (cluster 7) that involved 2 young MSM with sequences sampled in 2019. This subtype was circulated in MSM in France and was identified in the Philippines^19,20^. Only one case (0.1%) was detected in 927 samples collected between 1999 and 2012 in a nationwide study in Korea^6^. Both clustered individuals were recently diagnosed with HIV and had no history of sexual activity overseas. Therefore, we speculate that this subtype may have sporadic local transmission, possibly in MSM, in Korea.

We also found a mixed cluster harboring CRF07_BC (cluster 8) that involved 2 MSM and a heterosexual man^21–24^. Two non-clustered patients with CRF07_BC were Chinese MSM whose sequences were sampled in 2016 and 2018, respectively. CRF07_BC mainly circulated among IDUs in China in early 2000, spread from IDUs to their sexual partners and further to the general population in China, particularly after a dramatic increase in the MSM population since 2009^25^. In 2003, CRF07_BC was identified in Taiwan, with likely migration between 1998 and 2001, and has also become the predominant strain among IDU in Taiwan^26,27^. CRF07_BC has also spread to some countries in South East Asia that share borders with China, possibly through drug trafficking routes^27–29^. This subtype was also rarely reported in Korea, with a prevalence of 0.1% (1/927) in 1999–2012^6^. Considering that our sequences in the CRF07_BC clusters were sampled between 2016 and 2019, our results suggest that the CRF07_BC is circulating in local transmission chains, possibly between different risk groups in Korea. Therefore, a further nationwide study is needed.

The proportion of women among patients with non-B subtypes have fluctuated over time. The majority of women reported that they were infected through heterosexual contact with HIV-positive husbands or male sexual partners when they were not aware of their partner’s infection status. Their subtype distribution was mostly dependent on their partner’s subtypes. In the phylogenetic analysis, 28% (7/25) of women were included in 4 small clusters that belonged to CRF01_AE, with sizes ranging from 2 to 4 patients. Two clusters included only women and the remaining 2 were heterosexual male/female clusters. A male/female cluster included a married couple, an OW, and his partner, but epidemiological connections could not be identified in the other 3 clusters.

In OSs and their partners, CRF02_AG and A1, mainly circulating among African and non-Chinese Asian countries, were most commonly found^5,30^. In the 1980–1990s, many Korean OSs were workers employed at global shipping companies and were sent abroad as skilled workers; some of them were infected with HIV, particularly in Africa^31^. We found an A1 pair (cluster 6) containing 2 OSs. However, there was no epidemiological link between the 2 OSs. On the other hand, CRF_01AE accounted for more than 80% of OWs and their partners, which may be associated with the increasing numbers of OWs to Southeast Asia and China^30^.

Our findings should be considered in the context of several limitations. First, this was a hospital-based, retrospective, observational study. The collection of epidemiological information might have been limited because of the retrospective nature of our study. Therefore, we cannot rule out the presence of unmeasured confounding factors. Second, our study was conducted at a single center, and a small number of HIV cases were included. Therefore, the results should be generalized to other regions of the country with caution. Although the proportion of OS and their spouse were higher in this region at the beginning of epidemic, recent trends of increasing of MSM and decreasing age at HIV diagnosis are similar to those of nationwide study. During the study period, approximately 8.6% of the Korean population living with HIV infection visited the study hospital^2^. The southeastern region of Korea accounts for about 15% of all HIV infected patients in Korea^32^. Therefore, approximately 58% of HIV-infected patients in this region attended the study hospital and 43.2% of them were included for subtype analysis. Third, since the HIV-1 genotype resistance assay was routinely performed at the time of entry to care in our institution since 2011, some of the subjects treated before this time have not been included in our dataset, which might have resulted in selection bias. Many OSs were not included in the study due to death, transfer, or loss of follow-up before enrollment, or suppressed viral load due to ART at enrollment. In addition, selection bias was also possible because the sequences were only from persons engaged in clinical care who had an available genotype. Fourth, about 20% of the subjects were treatment-experienced patients, and their genotypic resistance tests were mostly performed when they returned to care after lost to follow-up, or some were performed at the time of treatment failure. Although there was no statistically significant difference in their proportion between subtype B group and non-B group, the patients who were diagnosed with HIV in the earlier years were more likely to be included in the treatment-experienced group, which might have resulted in selection bias. However, we included only treatment naïve patients in the phylogenetic cluster analysis. Fifth, Viroseq HIV-1 genotyping system used in this study is generally optimized for HIV-1 subtype B and has been used to sequence non-B subtypes with different genotyping sensitivities. It can also be affected by sensitivity of detection of different versions of the system^33,34^.

## Conclusions

In this study, despite the dominance of HIV-1 subtype B infection in the Korean HIV population, we found that the proportion of non-B infections has recently increased. Changes in epidemiological profiles have also influenced the distribution of HIV-1 subtypes in Korea. HIV-1 non-B subtypes are no longer limited to infections acquired abroad, such as those in OSs, and are spreading to multiple transmission groups, including MSM. Our results also highlight the continuous expansion of HIV-1 non-B subtype diversity, suggesting introducing new subtypes in certain risk groups or sexual linkages between different subgroups. Implementing continued HIV-1 molecular surveillance is essential for understanding and monitoring transmission cluster dynamics to improve HIV prevention efforts in Korea.

## Methods

### Study population

Among 1,197 patients with an HIV infection who visited the Pusan National University Hospital, Busan, Korea, from January 2005 to December 2019, 517 patients who underwent a genotypic antiretroviral resistance assay were enrolled in this study. Patients aged < 15 years at enrollment were excluded. Between 2005 and 2010, the genotypic resistance test was conducted primarily in patients failing therapy and partially in treatment-naive patients before starting ART. Since 2011, it has been routinely performed at the time of entry to care. The test was conducted in the study hospital from 2005 to 2016 and outsourced to Seoul Clinical Laboratories starting in 2017. Genotypic resistance tests were performed using the ViroSeq™ HIV-1 Genotyping System v2.0 (Abbott Laboratories, USA), and parts of the *pol* gene (protease/reverse transcriptase) were obtained. ViroSeq® HIV-1 Genotyping Software has been upgraded from v2.8 to v3.0 since 2016. In case a patient underwent multiple tests, the earliest sequence was used. Patients’ medical records were reviewed for epidemiological and clinical data. The study protocol was approved by the Institutional Review Board of Pusan National University Hospital (IRB number. H-2005-006-090). Our research was performed in accordance with the relevant guidelines and regulations. This study was retrospective and non-pharmacological, and the need for informed consent was waived. All phylogenetic and statistical analyses were performed on de-identified data-sets.

### HIV-1 subtype analysis

HIV-1 subtyping was determined using a combination of the following tools: REGA HIV-1 Subtyping Tool (version 3, http://regatools.med.kuleuven.be/typing/v3/typingtool, Rega Institute for Medical Research, Leuven, Belgium)^35^, COMET HIV-1 (https://comet.lih.lu, Luxembourg Institute of Health, Luxembourg)^36^, and jpHMM-HIV (http://jphmm.gobics.de/submission_hiv, Institute of Microbiology and Genetics, University of Gottingen, Niedersachsen Germany)^37^. Additionally, we used HIV BLAST (https://www.hiv.lanl.gov/content/sequence/BASIC_BLAST/basic_blast.html, Los Alamos National Laboratory, USA) to identify the nearest HIV-1 sequences from different geographical proximities. In the case of discordance between the various systems, we relied on the manual molecular phylogenetic analysis. The *pol* sequences were aligned with reference sequences of respective subtypes via ClustalW in BioEdit (version 7.2, Biological Sequence Alignment Editor, Raleigh, NC, USA). The 104 reference gene sequences of the subtypes were obtained from the HIV database (https://www.hiv.lanl.gov). A phylogenetic tree was constructed using the neighbor-joining method based on the Kimura 2-parameter model with 1000 bootstrap replicates in MEGA version 10.1.8 (https://www.megasoftware.net).

### Phylogenetic analysis of non-B subtypes

A maximum-likelihood phylogenetic tree that includes 83 sequences of ART-naive patients with non-B subtypes and 104 HIV-1 reference sequences were constructed via MEGA (version 10.1.8). Using MEGA, we selected the nucleotide substitution model most appropriate for analyzing our data set: the general-time reversible model with the proportion of invariable sites and gamma plus invariant sites distributed rate heterogeneity (GTR + G + I model). The reliability of the branching orders was assessed via a bootstrap analysis of 1000 replicates. We defined clusters as clades with high branch support values (> 90%) and short branch lengths (genetic distance < 0.015)^38^. All phylogenetic trees were visualized via FigTree version 1.4.4 (https://tree.bio.ed.ad.uk).

### Statistical analysis

Statistical analyses were performed with SPSS version 22.0 (IBM SPSS Statistics, USA). Categorical variables were compared using Pearson’s chi-squared test or Fisher’s exact test, and non-categorical variables were tested using the Mann–Whitney U-test or the Kruskal Wallis test. All tests of significance were two-tailed; *P* < 0.05 was considered significant.

## Data Availability

All data generated or analyzed during this study are included in this published article.
